# Synthesis of *Bis*(Isoxazol-4-Ylmethylsulfanyl)Alkanes and Some Metal Complexes as a Hepatoprotective Agents

**DOI:** 10.15171/apb.2018.031

**Published:** 2018-06-19

**Authors:** Vnira Rakhimovna Akhmetova, Rozalia Akramovna Galimova, Nail Salavatovich Akhmadiev, Albina Midkhatovna Galimova, Ravil Akhmetzyanovich Khisamutdinov, Galiya Maratovna Nurtdinova, Eduard Feliksovich Agletdinov, Valery Alekseevich Kataev

**Affiliations:** ^1^Institute of Petrochemistry and Catalysis, Russian Academy of Sciences, 141 Prospekt Oktyabrya, 450075 Ufa, Russia.; ^2^Bashkir State Medical University, 3 Lenin Str., 450008 Ufa, Russia.; ^3^Ufa Institute of Chemistry, Russian Academy of Sciences, 71 Prospekt Oktyabrya, 450054 Ufa, Russia.

**Keywords:** Metal-organic frameworks, Isoxazoles, Hepatoprotector, *In vivo*, Liver, Hepatitis

## Abstract

***Purpose:*** This research is devoted to designing the synthesis of sulfanyl-substituted 3,5-dimethylisoxazoles, which contain structural analogues of the SAM drug in the molecule. SAM (S-adenosyl-L-methionine), formed in the biosynthetic process, is used as an effective hepatoprotective drug. Complexation and hepatoprotective properties of the combinatorial series of bis(isoxazolylsulfanyl)ethane have been studied.

***Methods:*** Bis(isoxazol-4-ylmethylsulfanyl)alkanes were synthesized using the one-pot method. The structures of compounds were established by one-dimensional (^1^H,^13^C) and two-dimensional (COSY, HCQS, HMBC) NMR spectroscopy, mass-spectrometry and X-ray diffraction. The biological activity of the combinatorial series of sulfanyl derivatives of diketones, azoles and their metal complexes has been studied by in vivo method. Simulation of the animal associated processes was carried out in accordance with the principles of bioethics. Screening studies of hepatoprotective activity were carried out in a model of acute CC1_4_ intoxication after a single injection intraperitoneally as a 50% solution in olive oil. The pharmacologically known hepatoprotective drug SAM served as a control.

***Results:*** Two-step synthesis of novel α,ω-bis(isoxazol-4-ylmethylsulfanyl)alkanes was carried out via the multicomponent reaction between 2,4-pentandione, CH_2_O and α,ω-dithiols, then the resulting α,ω-bis(1,3-diketone-2-ylmethylsulfanyl)alkanes were transformed by hydroxyl amine to obtain bis-isoxasole derivatives. Promising precursor 1,2-bis(isoxazol-4-ylmethylsulfanyl)ethane was converted to metal complexes by interaction with PdCl_2_ or CuCl. The obtained compounds were found to be practically non-toxic compounds (1001 – 3000 mg/kg) according to the classification of K.K. Sidorov, but copper complex refers to low-toxic compounds substances (165 mg/kg). Compounds of sulfanyl ethane series demonstrate hepatoprotective activity.

***Conclusion:*** Palladium(II) complex being almost non-toxic possesses hepatoprotective activity comparable to the drug like SAM.

## Introduction

Currently the use of organometallic complexes in medicine is considered as an innovative approach, due to their unusual activity in biological systems.^1-3^ From the standpoint of metal-ligand homeostasis, organic complexes with essential metals forming part of the active site of many enzymes,^4-7^ are very promising for the treatment of pathological states.^8^ Recently^9^ it has been found that baicolin-copper complex is effective hepatoprotective agent unlike baicalin itself.

Breakthrough event was the discovery in the late 20th-century therapeutic properties of cisplatin,^10^ and other platinum complexes against cancer,^11,12^ which, unfortunately, have significant toxic side effects.^13,14^ Later it was shown that the less toxic palladium(II) complexes were also effective for the treatment of cancer. Nowadays there is the interest of researchers to look for low-toxic organometallic complexes with an effective anti-tumor^15-18^ or hepatoprotective activity.^19^

We have previously reported the one-pot effective synthesis of α,ω-*bis*(1,3-diketone-2-ylmethylsulfanyl)alkanes,^20^ which are promising precursors for methylsulfanyl substituted α,ω-*bis*-pyrazoles with pronounced inhibitory effects on alpha-amylase activity.^21-23^

Taking into account, that isoxazoles exhibit pharmacological properties,^24-27^ and sulfanyl substituted isoxazoles are polydentate ligands,^28,29^ our aim was to carry out the synthesis of novel complexes of Pd(II) and Cu(I) with 1,2-*bis*[(3,5-dimethylisoxazol-4-yl)methylsulfanyl] ligands and examine the toxicological and hepatoprotective properties of metallo-complexes and precursors thereof.

## Materials and Methods

### General procedures and materials 

The reaction products were characterized by ^1^H and ^13^C NMR spectra that were recorded on spectrometers Bruker Avance 400 NMR (400.13 MHz and 100.62 MHz) and Bruker Ascend III HD 500 (500.17 MHz and 125.78 MHz), internal standard TMS, solvent DMSO-d_6_. The homo- and heteronuclear 2D experiments were performed by the standard pulse sequences of Bruker. IR spectra were recorded on a Bruker Vertex-70V FTIR and Specord M80 spectrometers. Electrospray ionization (ESI) mass spectra were obtained on a HPLC massspectrometer LCMS-2010EV (Shimadzu) in positive and negative ions mode at the corona discharge needle ionizing electrode and ionizing capillary potential of – 3.5 kV. Sample solution (direct syringe sample inlet) under ESI conditions was in methanol (acetonitrile), mobile phase was acetonitrile/water, 95/5. Mass-spectra was recorded on a device MALDI TOF Autoflex III firm Bruker (compounds **2a-e**) with sinapinic acid as a matrix (see *Supplementary data*). Elemental analysis was performed on a Carlo Erba 1106 elemental analyzer. Melting points were determined on a Kofler hot-stage microscope and utilized uncorrected. Individuality and purity of synthesized compounds were controlled by means of TLC on Silufol UV-254 plates; I_2_ was used as developer.

#### General procedure of thiomethylation of 2,4-pentanedione with formaldehyde and a,ω-dithiols. 1,2-Bis[(pentane-2,4-dione-3-yl)methylsulfanyl]alkanes (***1a-e***)

In Schlenk vessel using a magnetic stir bar was added with formaldehyde (37% aqueous solution, 20 mmol, 1.47 mL) and α,ω-dithiol (10 mmol) stirred for 30 min in argon atmosphere. Then 2,4-pentanedione (20 mmol) and the promoter BuONa (10 mmol) in 5 mL CHCl_3_-C_2_H_5_OH (1:1) were added. The mixture was stirred for 1 h at r.t. The precipitate was filtered, washed with alcohol to give the target product **1a**: (81%, 2.58 g) as a white crystals, mp 139–141°C (data lit. 138–140°C). The spectra of other sulfanyl derivatives of bis-diketones are similar to those previously obtained.^21^

#### Synthesis of 1,2-bis[(3,5-dimethylisoxazol-4-yl)methylsulfanyl]alkanes (***2a-e*** *) (General method)*

Sulfanyl-substituted bis-diketones (10 mmol), 15 mL of ethanol were charged into the glass vessel, was added with small portions of hydroxylamine (25 mmol, 1.74 g). The reaction mixture was heated up to 60 °C and stirred for 2 h. Then formed precipitate was filtered, washed with water (2 × 15 mL), and dried in open air.

*1,2-Bis[(3,5-dimethylisoxazol-4-yl)methylsulfanyl]ethane* (**2a**) Yield: 97%; white crystals, mp: 155–156°C (data lit. 154-156°C^21^).

*1,3-Bis[(3,5-dimethylisoxazol-4-yl)methylsulfanyl]propane* (**2b**) white solide (56%): *R*_f_=0.59 (1:2:10 cyclohexane/CH_2_Cl_2_/EtOAc); mp 84–86 °С; IR (thin film) ν_max_ 1632, 1190, 1034, 886, 738 сm^-1^;^1^H NMR (DMSO-d_6_, 500 MHz) *δ*=3.53 (4Н, s, СH_2_); 2.48 (4Н, t, ^3^*J* = 7.2 Hz, H-9, -11); 2.33 (6Н, s, СH_3_); 2.20 (6Н, s, СH_3_); 1.77 (2Н, p, ^3^*J* = 7.2 Hz, СH_2_); ^13^C NMR (DMSO-d_6_, 125 МHz,) *δ*=166.1 (C, C-3, -15), 159.6 (C, C-5, -18), 111.3 (C, C-4, -14), 30.2 (CH_2_, C-9, -11), 28.9 (CH_2_, C-10), 22.8 (CH_2_, C-7, -13), 10.9 (CH_3_, C-21, -19), 10.1 (CH_3_, C-6, -20); MALDI TOF *m/z* 327.327 С_15_Н_23_N_2_O_2_S_2_ (calcd. 327.485); 349.267 С_15_Н_22_N_2_O_2_S_2_Na (calcd. 349.467); Anal. Calcd. for С_15_Н_22_N_2_O_2_S_2_: С, 55.18; Н, 6.79; N, 8.58; S, 19.64. Found: С, 55.34; Н, 6.85; N, 8.42; S, 19.71.

*1,4-Bis[(3,5-dimethylisoxazol-4-yl)methylsulfanyl]butane* (**2с**): white solide (94%): *R*_f_=0.61 (1:2:10 cyclohexane/CH_2_Cl_2_/EtOAc); mp 68–70 °С; IR (thin film) ν_max_ 1637, 1192, 1032, 893, 719 сm^-1^; ^1^H NMR (DMSO-d_6_, 500 MHz) *δ*=3.45 (4Н, s, СH_2_); 2.40 (4Н, m, СH_2_); 2.32 (6Н, s, СH_3_); 2.19 (6Н, s, СH_3_); 1.57 (4Н, m, СH_2_); ^13^C NMR (DMSO-d_6_, 125 МHz) *δ*=166.0 (C, C-3, -15), 159.7 (C, C-5, -19), 111.4 (C, C-4, -15), 30.7 (CH_2_, C-9, -12), 28.4 (CH_2_, C-10, -11), 22.7 (CH_2_, C-7, -14), 10.9 (CH_3_, C-20, -22), 10.1 (CH_3_, C-6, -21); MALDI TOF *m/z* 363.341 С_16_Н_24_N_2_O_2_S_2_Na (calcd. 363.494); Anal. Calcd. for С_16_Н_24_N_2_O_2_S_2_: С, 56.44; Н, 7.10; N, 8.23; S, 18.83. Found: С, 56.49; Н, 7.21; N, 8.19; S, 18.97.

*1,5-Bis[(3,5-dimethylisoxazol-4-yl)methylsulfanyl]-3-thiapentane* (**2d**): white solide (74%): *R*_f_= 0.66 (1:2:10 cyclohexane/CH_2_Cl_2_/EtOAc); mp 71–73 °С; IR (thin film) ν_max_ 3421 (N–H), 1633 (С=N), 1192 (С–N), 726 (С–S) сm^-1^; ^1^H NMR (DMSO-d_6_, 500 MHz) *δ*=3.61 (4Н, s, СH_2_); 2.74 – 2.61 (8H, m, SC_2_H_4_SC_2_H_4_S) 2.34 (6Н, s, СH_3_); 2.20 (6Н, s, СH_3_); ^13^C NMR (DMSO-d_6_, 125 МHz) *δ*=166.2 (C, C-3, -17), 159.7 (C, C-5, -20), 111.3 (C, C-4, -16), 31.6 (CH_2_, C-10, -12), 31.5 (CH_2_, C-9, -13), 22.7 (CH_2_, C-7, -15), 10.9 (CH_3_, C-21, -23), 10.1 (CH_3_, C-6, -22); MALDI TOF *m/z* 395.041 С_16_Н_24_N_2_О_2_S_3_Na (calcd. 395.558); 411.004 С_16_Н_24_N_2_О_2_S_3_K (calcd. 411.667); Anal. Calcd. for С_16_Н_24_N_2_О_2_S_3_: С, 51.58; Н, 6.49; N, 7.52 S, 25.82. Found: С, 51.67; Н, 6.53; N, 7.64; S, 26.13.

*1,6-Bis[(3,5-dimethylisoxazol-4-yl)methylsulfanyl]hexane* (**2e**): white solide (69%): *R*_f_= 0.59 (1:2:10 cyclohexanes/CH_2_Cl_2_/EtOAc); mp 76–78 °С; IR (thin film) ν_max_ 3436, 1634, 1196, 1038, 721 сm^-1^; ^1^H NMR (DMSO-d_6_, 500 MHz) *δ*=3.52 (4Н, s, СH_2_); 2.39 (4H, t, ^3^*J* = 7.2 Hz, CH_2_S); 2.33 (6H, s, CH_3_); 2.20 (6H, s, CH_3_); 1.49 (4Н, m, СH_2_); 1.30 (4Н, m, СH_2_); ^13^C NMR (DMSO-d_6_, 125 МHz) *δ*=165.9 (C, C-3, -18), 159.6 (C, C-5, -21), 111.4 (C, C-4, -17), 31.1 (CH_2_, C-9, -14), 29.2 (CH_2_, C-10, -13), 28.3 (CH_2_, C-11, -12), 22.8 (CH_2_, C-7, -16), 10.9 (CH_3_, C-22, -24); 10.1 (CH_3_, C-6, 23); MALDI TOF *m/z* calculated for 369.316 С_18_Н_29_N_2_O_2_S_2_ (calcd. 369.565); С_18_Н_28_N_2_S_2_Na 391.267 (calcd. 391.547); 407.222 С_18_Н_28_N_2_S_2_K (calcd. 407.655); Anal. Calcd. for С_18_Н_28_N_2_S_2_: С, 58.66; Н, 7.66; N, 7.60; S, 17.40. Found: С, 58.87; Н, 7.81; N, 7.54; S, 17.62.

#### *Сis-S,S-dichloride-1,6-(3,5-dimethylisoxazol-4-yl)-2,5-dithiahexane palladium(II) complex (***3**)

In the glass vessel (1.405 mmol, 0.25 g) palladium(II) chloride was dissolved in 15 mL of acetonitrile by stirring at 60 °C. After cooling up to r.t. 1,2-*bis*[(3,5-dimethylisoxazol-4-yl)methylsulfanil]ethane (1.405 mmol, 0.44 g) was added and reaction mixture was stirred for 3 h. The resulting bright yellow precipitate was filtered through filter paper (blue ribbon) and washed by acetonitrile, water and dried in open air with formation yellow powder **3** (54%); mp > 250°С (dec.); IR (thin film) ν_max_ 1635 (br), 1274, 1250, 1193, 883, 829, 715, 661, 334, 307 cm^-1^; ^1^H NMR (DMSO-d_6_, 500 MHz) of diastereomeric mixture (AB system):* δ*=4.63 (2H_a_, dd, ^3^*J* 14.4, Hz, CH_2_), 4.33 (2H_a_, dd, ^3^*J* 14.4 Hz, IzCH_2_S), 4.47 (2H_b_, dd, ^3^*J* 14.0 Hz, CH_2_), 4.18 (2H_b_, dd, ^3^*J* 14.0 Hz, CH_2_), 3.51 (2Н_a_, dd, ^2^*J =* 9.2 Hz, SCH_2_CH_2_S), 3.15 and 3.09 (2H_b_, br s, SCH_2_CH_2_S), 2.93 (2Н_a_, dd, ^2^*J =* 9.2 Hz, SCH_2_CH_2_S), 2.43 (6Н, s, СH_3_), 2.26 (6Н, s, СH_3_); ^13^C NMR (DMSO-d_6_, 125 МHz) *δ*=168.9 (C, C-3, -16), 159.6 (C, C-5, -19), 107.8 (C-4, -15), 37.5 and 37.3 (CH_2_, C-9, -10), 30.4 and 29.7 (CH_2_, C-7, -14), 11.5 (CH_3_, C-13,23), 10.3 (CH_3_, C-6,22); ESI *m/z* 527 [*M*+Cl]- (100); Anal. Calcd. for С_14_Н_20_Сl_2_N_2_O_2_PdS_2_: С, 34.33; Н, 4.12; Cl, 14.48; N, 5.72; Pd, 21.73, S, 13.09. Found: С, 34.07; Н, 3.84; Cl, 14.74; N, 5.82; Pd, 22.12, S, 13.10.

#### Сis-S,S-dichloride-1,6-(3,5-dimethylisoxazol-4-yl)-2,5-dithiahexane copper(I) complex (***4***)

In a three-necked flask equipped with thermometer, refluxer and argon was charged with 1,2-bis[(3,5-dimethylisoxazol-4-yl)methylsulfanyl]ethane in 4 mL CH3CN 0.28 g (2.81 mmol) and copper(I) chloride was added (1.405 mmol, 0.44 g). Than reaction mixture was stirred for 3 h at 60 °C. The resulting white precipitate was filtered through filter paper (blue ribbon) and washed with acetonitrile. The complex **4** was received as white powder (0.43 g, 60%); mp 221–223 °С; IR (thin film): ν 1629, 1196, 884, 724, 480, 398, 320. ^1^H NMR (DMSO-d_6_, 500 MHz) *δ*=3.53 (4Н, s, СH_2_), 2.57 (4Н, m, SC_2_H_4_S), 2.24 (6Н, s, СH_3_), 2.10 (6Н, s, СH_3_); ^13^C NMR (DMSO-d_6_, 125 МHz) *δ*=166.0 (C, C-3, -19), 160.1 (C, C-5, -22), 111.5 (C, C-4, -18), 31.2 (CH_2_, C-9, -10), 22.8 (CH_2_, C-7, -17), 11.0 (CH_3_, C-16, -24), 10.2 (CH_3_, C-6, -23). Anal. Calcd. for С_14_Н_20_N_2_O_2_S_2_Cu_2_Сl_2_: С, 32.94; Н, 3.95; Cl, 13.89; Cu, 24.90; N, 5.49; S, 12.56. Found, %: С, 32.58; Н, 3.82; Cl, 13.51; Cu, 24.78; N, 5.43; S, 12.87.

### Crystal Structure Determination and Refinement

The X-ray diffraction experiments of **2c** and **2d** single-crystals were carried out by a Bruker SMART 1000 CCD area detector using graphite monochromated MoeKa radiation at 100 K. All calculations were performed on an IBM PC/AT using the SHELXTL software Atomic coordinates, bond lengths, bond angles, and thermal parameters have been deposited at the Cambridge Crystallographic Data Centre (CCDC). X-ray diffraction data of **2c** and **2d** single-crystal was collected on a XCalibur Eos diffractometer with graphite monochromated Mo-Kα radiation (λ=0.71073 Å). Collection and processing of data performed with using the program CrysAlis^Pro^ Oxford Diffraction Ltd., Version 1.171.36.20. The structure was solved by direct methods as implemented in the program SHELXS-97.^30,31^ The refinement was carried out using SHELXL-97. The structure was refined by a fullmatrix least-square technique using anisotropic thermal parameters for non-hydrogen atoms and a riding model for hydrogen atoms.

Crystallographic data for the structure of **2c** have been deposited in the Cambridge Crystallographic Data Centre as a CIF deposition with file number CCDC 1545010. Copies of these data can be obtained free of change on application to CCDC, 12, Union Road, Cambridge, CB2 1EZ, UK (fax: 44 1223 336033, e-mail: deposit@ccdc.cam.ac.uk) or from http://www.ccdc.cam.ac.uk/data_request/cif.

Crystallographic data for the structure of **2d** have been deposited in the Cambridge Crystallographic Data Centre as a CIF deposition with file number CCDC 1545008. Copies of these data can be obtained free of change on application to CCDC, 12, Union Road, Cambridge, CB2 1EZ, UK (fax: 44 1223 336033, e-mail: deposit@ccdc.cam.ac.uk) or from http://www.ccdc.cam.ac.uk/data_request/cif.

### Biological assay

Simulation of animal-related processes was carried out in accordance with rules of laboratory practice (GLP) and the ethical norms of the Geneva Convention (1971). Conditions of experiment and keeping animals were carried out according to modern requirements.^32^ The tissue was fixed in a 10% solution of neutral formalin for light-optical examination of the liver. For further histological treatment it was used samples of 5-7 mm thickness which were cut from a large proportion of the liver by cross-sectional dissection and subjected to standard treatment on the histological complex MICROM (Carl Zeiss, Germany). Samples were dehydrated in alcohols with increasing concentration, followed by pouring into paraffin blocks.

The studies were carried out on mice with a line BALB/CJ weighing 20 – 23 g (mice are provided by the Bashkir State Medical University Vivarium, Ufa, Russia). The animals were kept in 10 cells in a cage in standard vivarium conditions at an air temperature of 18 – 22 °C and a relative humidity of 50 to 65%. During the process there were free access to water and feed (~ 5 g/day).

## Results and Discussion

### Chemistry

**Figure 1:**
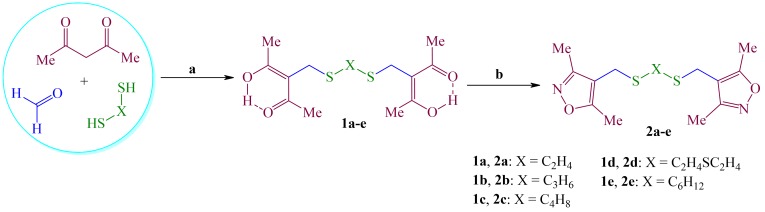
Reagents and conditions: a) BuONa, RT, 1.5 h, C_2_H_5_OH:CHCl_3_ (1:1); b) NH_2_-OH · HCl, 70 ^o^C, C_2_H_5_OH

The key substrates for the three step synthesis of target metallo-complexes of Pd(II) and Cu(I) was α,ω-*bis*[(pentane-2,4-dione-3-yl)methylsulfanyl]alkanes **1a-e** produced by a *n*-BuONa *mediated* multicomponent reaction (MCR) between 2,4-pentanedion, CH_2_O and α,ω-dithiols.^20^ The yield of products **1a-e** was decreased with increasing the aliphatic chain of α,ω-dithiols from 97 to 54%. Substrates **1a-e** was successfully converted into α,ω-*bis*[sulfanylmethyl(3,5-methylisoxazol-4-yl)]alkanes **2a-e** with high yields through the interaction between **1a-e** and hydroxylamine hydrochloride in refluxing ethanol during 2 h (Figure 1).

It should be noted, that in the indicated above two-step sequence, the total yield of **2a** is higher than in the case of *one-pot* four-component reaction between 2,4-pentandione, CH_2_O, 1,2-ethanedithiol and NH_2_OH·HCl.^21^

**Figure 2:**
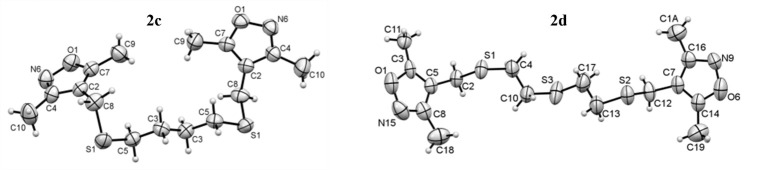
The geometry of molecules of compounds **2c** and **2f** in a crystal. Atoms are represented by thermal ellipsoids (*p* = 50%).

According to X-ray data (Figure 2), compound **2c** is crystallized in the monoclinic and **2d** - in orthorhombic crystalline system. It was found that *bis*(3,5-dimethylizoxazol) rings are in the *cis*-conformation with respect to S-(C)_n_-S fragment for compound **2c** and *trans*-configuration for compound **2d**. The crystallographic data for compounds **2с** and **2d** are collected in Table 1.

Among the synthesized 1,2-*bis*[sulfanylmethyl(3,5-dimethylisoxazol-4-yl)]alkanes **2a-e, 2a** is the most promising for practical application taking into account the production efficiency and availability of the starting reagents.

**Figure 3:**
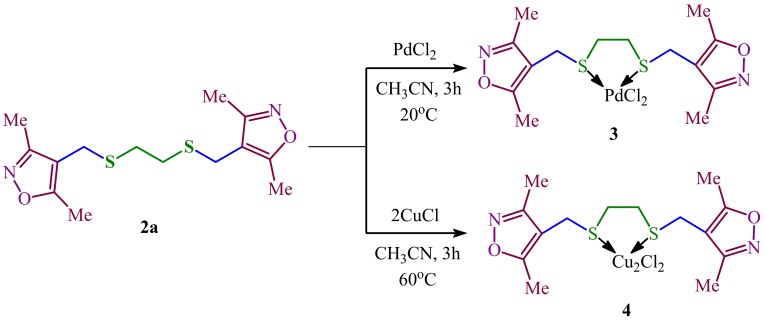
Reagents and conditions: a) PdCl_2_, CH_3_CN, 20 °C, 3h; b) СuCl, CH_3_CN, 60 °C, 3h.

So that, 1,2-*bis*[sulfanylmethyl(3,5-dimethylisoxazol-4-yl)]ethane **2a** was then transformed to Pd(II) complex **3** by reaction with PdCl_2_ in CH_3_CN. According NMR analysis, Pd(II) complex **3** in solution is the mixture of diastereomers (see *Supplementary data*). Using CuCl under the same reaction conditions Cu(I) complex **4** was also produced in 97% yield (Figure 3). According elemental analysis for complex **3** the ligand-metal ratio was 1:1 and for 4 as 1:2. In IR spectra of complexes **3** and** 4** there are signals of M-S: bonds is Pd-S in region 334 cm^-1^ and Cu-S in region 320 cm^-1^

### Biology

Combinatorial series of compounds **1a**, **2a**, **3**, and **4** having the same alkylsulfanyl chain and different cyclic fragments of substitutes were assessed as hepatoprotective agents. They were used as solutions in TWIN oil.

**Figure 4:**
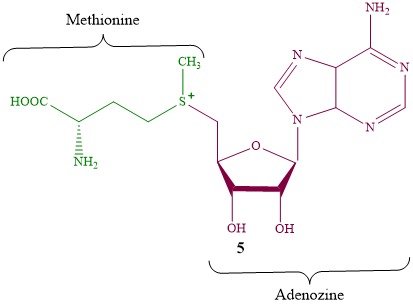
Structure of S-adenosyl-L-methionine 5

As known, the SAM drug (S-adenosyl-L-methionine **5**, Figure 4) being produced via biosynthetic process is used for treatment of large group of diseases associated with the hepatotoxic action of the chemicals or the alcohol causing morphological changes in liver tissue, metabolism disorder or the toxic liver damages^33-35^ SAM drug is considered as non-toxic sulfanyl-containing hepatoprotectors.^36^ It provides a stability of hepatocytes.^37^ Moreover, the transsulfuration (synthesis and turnover of glutathione and taurine, as well as conjugation and detoxication of bile acids and other xenobiotics), aminoproliliration and transmethylation processes are activated.

Obviously SAM is a powerful antioxidant due to its sulfur atoms and heterocyclic fragments in the structure.^38,39^ As seen, compounds **2a-e, 3, 4** also contain these units.

Thus, we have used SAM as the object of comparison to study toxicity and hepatoprotective activity of the compounds **1a**, **2a**, **3** and **4**.

**Table 1:** Crystallographic and structure refinement data for 2c and 2d

**Compounds**	**2c**	**2d**
Empirical formula	C_16_H_24_N_2_O_2_S_2_	C_16_H_24_N_2_O_2_S_3_
Formula weight	340.49	372.55
T/K	298	298
Crystal system	orthorhombic	monoclinic
Space group	Pbcn	P2_1_/c
a/Å	17.514(2)	5.0140(7)
b/Å	7.8852(9)	11.6177(7)
c/Å	13.027(2)	33.308(9)
α/°	90	90
β/°	90	93.45(2)
γ/°	90	90
V/Å^3^	1799.1(4)	1936.7(6)
Z	4	4
ρ_calc_mg/cm^3^	1.257	1.278
μ/mm^-1^	0.304	0.392
F(000)	728.0	792.0
Crystal size/mm^3^	0.54 × 0.26 × 0.22	0.71 × 0.30 × 0.28
2Θ range for data collection	4.66 to 62.82°	6.03 to 62.04°
Index ranges	-25 ≤ h ≤ 22 -11 ≤ k ≤ 10 -18 ≤ l ≤ 17	-7 ≤ h ≤ 3 -16 ≤ k ≤ 15 -36 ≤ l ≤ 29
Reflections collected	9025	4900
Independent reflections	2712[R_int_ = 0.0737, R_sigma_ = 0.0479]	3079[R_int_ = 0.0198, R_sigma_ = 0.0331]
Data/restraints/parameters	2712/0/126	3079/0/212
Goodness-of-fit on F^2^	1.057	1.050
Final R indexes [I>=2σ (I)]	R_1_ = 0.0570, wR_2_ = 0.1524	R_1_ = 0.0744, wR_2_ = 0.1750
Final R indexes [all data]	R_1_ = 0.0898, wR_2_ = 0.1934	R_1_ = 0.1034, wR_2_ = 0.1943
Largest diff. peak/hole / e Å^-3^	0.32/-0.29	0.40/-0.22

### Parameters of acute toxicity

To get reliable results acute toxicity was determined with Litchfield and Wilcoxon method modified by Prozorovskiy.^40^ As a result of determining the comparative evaluation of acute toxicity in albino mice after intraperitoneal injection and oral administration, it was established that compounds **1a**, **2a** and **3** are assigned to the group of virtually non-toxic compounds (1001 – 3000 mg/kg) according to the Sidorov classification (Table 2).^40^ After oral administration, LD_50_ value does not differ essentially from those of intraperitoneal administration.

Thus, structure-activity relationship shows that sulphanyl *bis*-diketone **1a** is less toxical compound (Sidorov classification, non-toxic group). It is not trivial fact that Pd(II) *cis*-chelate S,S-complex **3** also refers to group being not toxic. As seen from the Table 1, Cu(I) *cis*-chelate S,S-complex **4** is more toxical compound (Sidorov classification, low-toxic group). For this reason, compound **4** is not promising to treat liver diseases.

### The model of acute hepatitis

Screening studies of compounds **1a** and** 3** were carried out on a model of acute toxicity *in vivo*. Simulation of animal-related processes was carried out with the principles of bioethics. The animals received single dose of CCl_4_ 0.2 mL/kg intraperitoneally as a 50 percent solution of olive oil. The compound was administered intraperitoneally in a dose of 25 mg/kg 1 hour before the injection of CCl_4_. As control was used SAM **5** (ademetionine), a pharmacologically known hepatoprotective drug in a dose of 25 mg/kg. The control group received 0.2 mL of saline solution (Table 3).

Thus, compound **3** in dose of 25 mg/kg has a strong antitoxic effect on the model of acute intoxication of CCl_4_. In other words the compound 3 in the dose of 25 mg/kg causes significant lowering of lethality from 50 percent to 0 percent, compared with untreated animals in control group.

Biochemical analysis of blood were taken on the 10-th day of observation of acute hepatitis, caused with CCl_4_, to control the development of cytolytic syndrome and evaluate degree of liver injury.

The complex **3** at dose 25 mg/kg resulted in a significant lowering (p<0.05) alanine aminotransferase by 70% comparing with untreated group of animals. At the same time the reference preparation ademetionine at the same dose resulted a significant lowering only by 53% (p<0.05) (Table 4).

Compounds **1a** and **2a** are less active according to this indicator. A significant lowering of aspartate aminotransferase between control and experimental groups was not recorded. By the 10 day of observation in all groups with an acute hepatitis the level of conjugated bilirubin raised. Compared to the intact group of animals figures receiving compound **3** – by 20.2%, **2a** – at 55.9%, **1a** – 58.8%, SAM – 25.9% in the group with the drug **3** - less often.

**Table 2:**
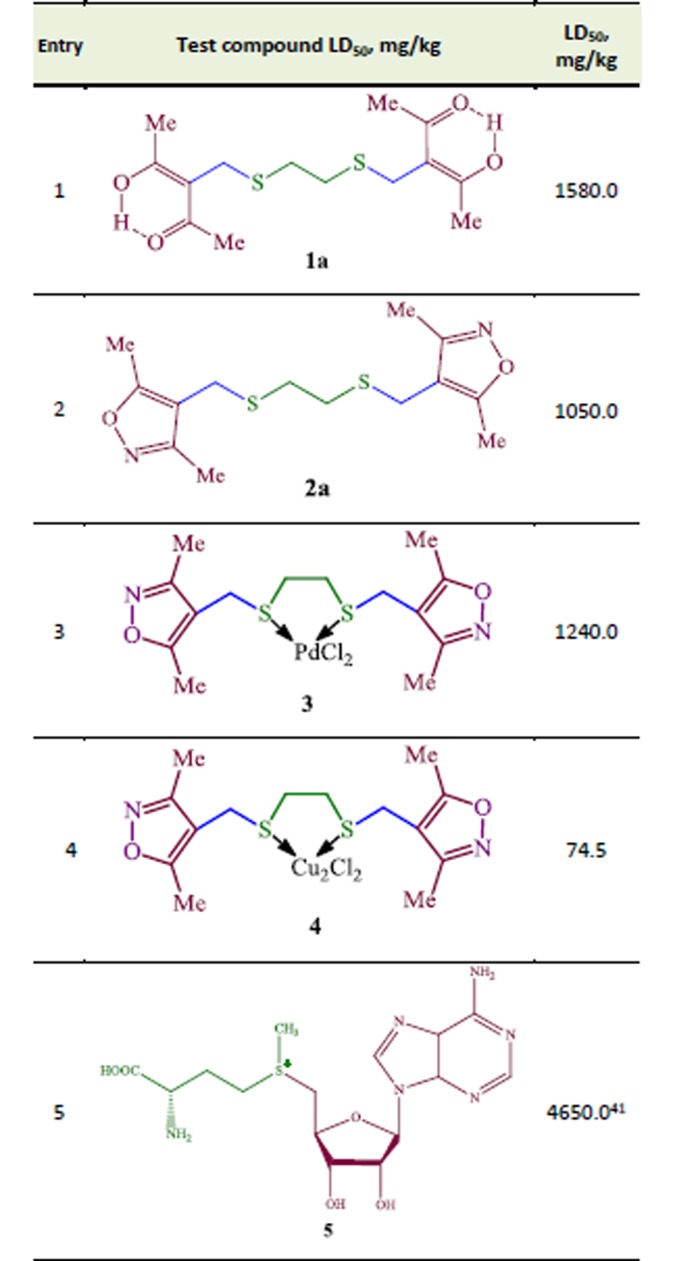
Acute toxicity study of derivatives of 1,2-bis[(pentane-2,4-dione-3-yl)methylsulfanyl]}ethane

**Table 3:** Effects of compounds **1a, 2a, 3** and SAM **5** survival of white mice with acute toxic hepatitis

**Administering compounds **	**Number of animals in groups**	**Surviaval rate on the 10 day of observation, %**
Saline solution 0.2 mL/kg (intact group)	10	100
CCl_4_ 0.2 mL/kg (control group)	10	50
SAM 25 mg/kg + CCl_4_ 0.2 mg/kg	10	80
**1a** 25 mg/kg + CCl_4_ 0.2 mg/kg	10	70
**2a** 25 mg/kg + CCl_4_ 0.2 mg/kg	10	60
**3** 25 mg/kg + CCl_4_ 0.2 mg/kg	10	100

**Table 4:** Effects of compounds **1a, 2a, 3** and SAM on indicators AST, ALT, and direct bilirubin serum white mice with acute toxic hepatitis

**Groups of animals**	**ALT,** **mcmol/mL/h**	**АSТ,** **mcmol/mL/h**	**Bilirubin direct serum, mcmol/L**
Control intact	1.49 ± 0.22	1.09 ± 0.07	8.3 ± 2.2
Control (CCl_4_)	4.72 ± 0.57*	1.71 ± 0.14*	19.5 ± 5.1*
SAM+ CCl_4_	2.21 ± 0.21	0.92 ± 0.06	11.2 ± 3.2
**3**+ CCl_4_	1.51 ± 0.31**	0.98 ± 0.02**	10.4 ± 2.6**
**2a**+ CCl_4_	2.32 ± 0.48**	0.94 ± 0.06**	20.1 ± 4.5
**1a**+ CCl_4_	2.68 ± 0.64	0.93 ± 0.07	18.8 ± 5.1

Note: * - significant differences between indicators of intact animals, ** - significant differences from that of group CCl_4_

### Histological examination

Staining with hematoxylin, eosin, by standard methods on histological complex MICROM. Histological activity index (HAI) was defined. Evaluation System indicators protein dystrophy, inflammatory infiltration, hyaline drop dystrophy - a 4-point scale.

Administering CCl_4_ without treatment led to gross structural changes in the form of large-drop dystrophy of hepatocytes, lymphohistiocytic infiltration of the liver structure.

Compounds pretreatment and heptral at dose 25 mg/kg led to less expression of morphological changes of liver structures: reduction of inflammatory infiltration, necrosis of hepatocytes, hepatocyte degeneration reduction degree.

Semi-quantitative method for assessing the degree of activity of pathological processes in the liver showed:

1. Significant reduction in HAI compared with results of control group was observed during therapy with Compound 3 and heptral. Compounds **1a** and **2a** had no significant digits.
2. The degree of fatty liver among white mice, treated with Compound **3** was minimal, hepatocytes with fatty inclusions are located only on the periphery of the hepatic lobule. Other animal groups **1a** and **2a** had moderate degree – 1/3 – 1/4 the length of the hepatic beams, hearths cirrhosis, liver tissue was sealed.

A new sensibly nontoxic (IV class) compound *cis*-S,S-dichloride-1,6-(3,5-dimethylisoxazol-4-yl)-2,5-dithiahexane palladium(II) complex **3** with hepatoprotective activity in laboratory animals (white mice) at a dose of 25 mg/kg intraperitoneally on acute hepatitis model induced by carbon tetrachloride was discovered. Compound **3** exeled the reference preparation ademetionine (SAM) for indications:

- animal survival (100%, SAM – 80%);
- biochemical (ALT, AST, bilirubin direct) parameters;
- histological (liver parenchyma lesions are minimal) parameters.

On the basis of biochemical tests (ALT, AST, bilirubin) and histological compounds displayed hepatoprotective activity which decreased in the number of **3** ˃ **1a** ˃ **2a**.

## Conclusion

In summary, we have developed a two step effective synthesis of α,ω-*bi*s(3,5-dimethylisoxazol-4-ylmethylsulfanyl)alkanes via the interaction between 2,4-pentandione, CH_2_O, α,ω-dithiols and next with hydroxyl amine. It was shown, that new Pd(II) and Cu(I) complexes are efficiently formed when using 1,2-*bi*s(isoxazol-4-ylmethylsulfanyl)ethane as ligand. The *in vivo* method has demonstrated*,* that combinatorial row - 1,2-*bis*[(pentane-2,4-dione-3-yl)methylsulfanyl]ethane **1a**, 1,2-*bis*[sulfanylmethyl(3,5-dimethylisoxazol-4-yl)]ethane **2a** and its complex with PdCl_2_ dichlorodi(3,5-dimethylisoxazol-4-yl)-1,2-dithiaethane palladium(II)) **3** are virtally non-toxic and exhibit hepatoprotective activity. The leader among them is palladium(II) complex dichlorodi(3,5-dimethylisoxazol-4-yl)-1,2-dithiaethane **3**, whose activity is comparable to SAM.

## Acknowledgments

This work was partially financially supported by the Grant of the republic of Bashkortostan young scientists and youth research teams. The reported study was funded by Russian Foundation for Basic Research and Academy of Sciences of the Republic of Bashkortostan according to the research project № 17-43-020292 p_a and project part 4.6007.2017/8.9. Structural studies of the compounds obtained were performed using unique equipment in "Agidel" collective usage centre (state assignment *АААА-А17-117012610060-7*).

## Ethical Issues

The study was carried out under ethical principles. Permission from the Local Ethics Committee of Bashkir state medical university is presented in supporting information.

## Conflict of Interest

The authors declare that they have no conflict of interest.
